# Short-Term Degradation of Bi-Component Electrospun Fibers: Qualitative and Quantitative Evaluations via AFM Analysis

**DOI:** 10.3390/jfb9020027

**Published:** 2018-03-30

**Authors:** Marica Marrese, Valentina Cirillo, Vincenzo Guarino, Luigi Ambrosio

**Affiliations:** Institute for Polymers, Composites and Biomaterials, National Research Council of Italy, 80125 Naples, Italy; m.marrese@vu.nl (M.M.); valentina.cirillo@unina.it (V.C.); ambrosio@unina.it (L.A.)

**Keywords:** atomic force microscopy (AFM), roughness, force spectroscopy, bi-component electrospun fibers, in vitro degradation studies

## Abstract

Electrospun polymeric fibers are currently used as 3D models for in vitro applications in biomedical areas, i.e., tissue engineering, cell and drug delivery. The high customization of the electrospinning process offers numerous opportunities to manipulate and control surface area, fiber diameter, and fiber density to evaluate the response of cells under different morphological and/or biochemical stimuli. The aim of this study was to investigate—via atomic force microscopy (AFM)—the chemical and morphological changes in bi-component electrospun fibers (BEFs) during the in vitro degradation process using a biological medium. BEFs were fabricated by electrospinning a mixture of synthetic-polycaprolactone (PCL)-and natural polymers (gelatin) into a binary solution. During the hydrolytic degradation of protein, no significant remarkable effects were recognized in terms of fiber integrity. However, increases in surface roughness as well as a decrease in fiber diameter as a function of the degradation conditions were detected. We suggest that morphological and chemical changes due to the local release of gelatin positively influence cell behavior in culture, in terms of cell adhesion and spreading, thus working to mimic the native microenvironment of natural tissues.

## 1. Introduction

Recent advances in micro- and nano-manufacturing technologies offer the chance to design instructive scaffolds with a highly defined and controllable morphology, able to reproduce the microstructural organization of cells in native tissues [1]. Scaffolds have to resemble the physical structure of native extracellular matrix (ECM) of tissues, mainly organizing cells in three-dimensional networks and releasing growth factors, thus providing an architecture on which cells might adhere, and developing new functional tissues to be implanted into the desired organ site. First of all, a scaffold must be able to respond to specific mechanical stresses depending on the particular properties of tissues to be regenerated [2,3]. Additional factors to be considered in the design of scaffolds are related to the porosity features, mainly deputed to address the release mechanisms of nutrients and growth factors, mandatory to properly trigger cell activities [4,5]. 

In this context, the biomaterial plays a key role and it has to be accurately selected depending upon the definite properties of tissues and their specific functions in vitro. The main characteristics for biomaterials are biocompatibility, to avoid unwanted response of the organism to the implant, and biodegradability, so as to be reabsorbed simultaneously with tissue formation [6]. In the past, metal and ceramic implants have been widely used in the biomedical field, especially in the orthopedic field. However, they have revealed two major drawbacks for applications in tissue engineering. Firstly, they are not biodegradable, and secondly their workability is very limited. 

For this reason, polymers from natural sources have received a growing interest from the scientific community. Natural polymers such as collagen [7,8], glycosaminoglycans [9,10], hyaluronan derivatives [11], chitin, and chitosan [12,13] have been successfully used to repair nerves, skin, cartilage, and bones due to their innate ability to mimic the cell’s microenvironment. However, their structural weakness and poor mechanical performance currently represent the main shortcomings in scaffolds manufacturing. To overcome these limitations, they may be combined with synthetic polymers with biodegradable properties such as PCL, which degrades extremely slowly by hydrolysis, showing low values of water absorption and weight loss after 110 weeks in aqueous medium simulating body fluids as widely documented in the literature [14,15].

However, the choice of an appropriate processing technique to fabricate scaffolds made of natural and synthetic polymers—i.e., bi-component scaffolds—is far from easy, due to the strong difference in terms of chemical/physical properties of single materials and their different behavior under the applied process conditions (i.e., solvent interaction, temperature effect, etc.). 

Currently, electrospinning is emerging as a relevant processing technique to produce micro- and nano-structured bi-component fibrous scaffolds [16,17]. An accurate design of electrospinning process conditions may assure a fine control of spatial orientation of fibers and patterning into the scaffolds, thus forming interconnected porous systems able to mimic the architecture of the ECM, present in both hard [18] and soft tissues [19]. 

A big challenge currently consists in the identification of new methodologies to more deeply characterize the response of cells in vitro, collecting more information at different size scale—from tissue to macromolecular level. In this context, atomic force microscope (AFM) may be a valid tool for characterizing surface topography and understanding the specific properties (mechanical, chemical and physical) of biomaterials [20]. AFM is a high-resolution probe-scanning microscope (SPM) based on short-range interactions between a small tip and a sample. The image processing is similar for this family of microscopes; the main differences reside in the probe and the corresponding interaction with the sample. More in detail, the working principle is related to the interaction forces between different atoms as a function of the reciprocal distance [21,22,23]. Unlike “classic” microscopes, like optical or electronic, AFM bases its magnifying effect on a principle different from that of the interaction of light or electrons with matter. AFM allows for morphological analysis of biological materials at resolutions not achievable by optical microscopy. In addition to information, strictly necessary to generate the topographic map, with AFM, it is possible to fully characterize micro- and nanostructured scaffolds in terms of biomaterials performance, processes, physical, chemical, and mechanical properties [24,25]. Though bi-component scaffolds have been widely investigated in vitro in terms of biocompatibility and cell materials interactions, only recently some studies have focused upon the in vitro role of gelatin release from bi-component fibers on cell activities [26].

Herein, we proposed a novel approach to study the in vitro degradation of bi-component electrospun fibers (BEFs) obtained by the combination of synthetic (PCL) and natural polymers (gelatin). In particular, this study aimed at investigating how the peculiar chemical and morphological changes in BEFs occurring during the degradation process in simulated culture medium and in phosphate-buffered solution (PBS) might be detected by optimizing the use of AFM techniques. Hence, we demonstrated that scaffold exposure to common aqueous in vitro medium differently modify the fiber microstructure and morphology, due to different protein interactions occurring during the in vitro degradation. This experimental result could be adopted to design innovative in vitro models for the investigation of undiscovered biological mechanisms involved in cell material interactions in the perspective of preclinical evaluations.

## 2. Results

**Figure 1:**
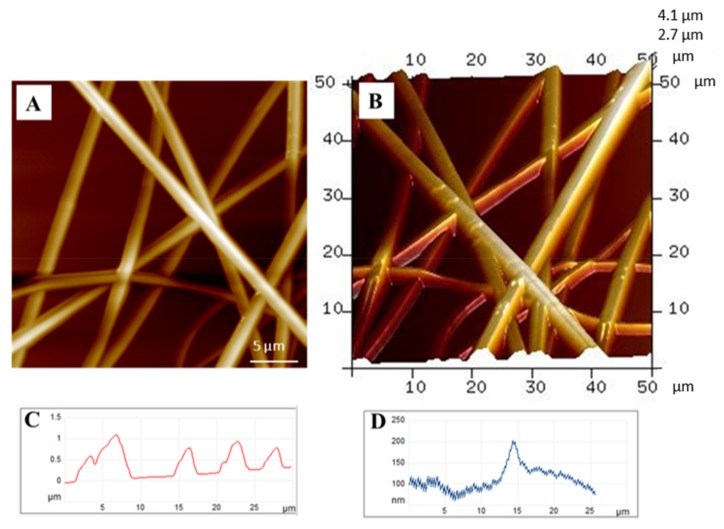
Morphology of electrospun scaffolds: (**A**) 2D and (**B**) 3D image of atomic force microscopy (AFM) analysis at a 5 µm scale; (**C**,**D**) Cross-section analyses across and along bi-component electrospun fiber (BEF) scaffolds.

BEFs were cultured up to 9 days to study via AFM the effect of degradation of gelatin in aqueous medium on the morphology of proposed scaffolds. Electrospun fibers were directly collected on glass slides to investigate fiber morphology in a simulated in vitro culture. Process parameters were properly set to obtain microfibers without defects as described in the Materials and Method section. 2D and 3D images of untreated samples—used as controls—were used as a reference (Figure 1A,B). Tapping mode (TM)-AFM measurements, performed directly on the fibers surface, revealed a roughly circular section of fibers with a diameter of 2.38 ± 0.5 μm (Figure 1C,D). Characteristic dimensions (i.e., diameter and thickness) of the fibers were measured on the AFM height images through section analysis. Average fiber diameter, calculated on 10 measurements taken from different sample images (*n* = 5), was 2.38 ± 0.5 μm for the control (CTR) sample. The cross-section of the fiber surface showed a flattened profile, as confirmed by the thickness value of about 200 nm respect to the diameter. The interaction with the simulated culture media led to a change in fiber morphology, with differences in the kinetics of scaffolds degradation as a function of the cell culture media, thus addressing cell adhesion and proliferation.

**Figure 2:**
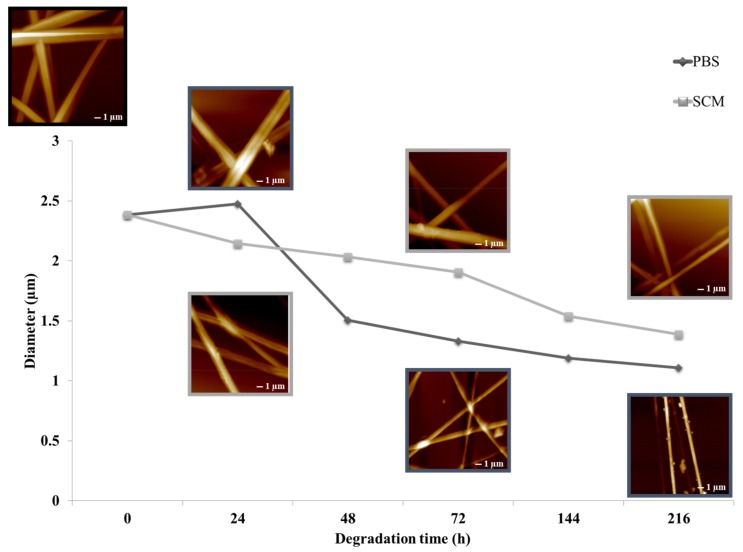
Degradation study of bi-component electrospun scaffolds via AFM analysis. Variations in fiber diameter after 1, 2, 3, 6, and 9 days of incubation in phosphate-buffered solution and simulated culture medium (SCM) are reported.

**Figure 3:**
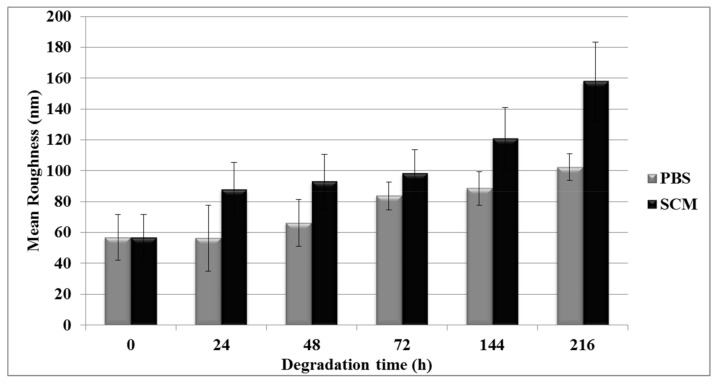
Quantitative AFM analysis: mean roughness Rq (mean ± SD) of BEF surfaces incubated in PBS and SCM.

The AFM morphological analysis of fibers surface did not appear to be altered after nine days of incubation; however, as a consequence of gelatin loss, irregular topographical features were detected on the fiber surface as shown in the topography image (Figure 2). By processing these data, a reduction in BEF diameter to 1.38 ± 0.37 μm was found in the CTR sample after nine days of treatment in simulated culture medium (SCM). Moreover, in the PBS, the reduction in mean diameter was more evident—after nine days, the diameter was 1.10 ± 0.29 μm. As expected, the SCM media, compared with the PBS, did not visibly degrade the fibers, but a slight swelling of the overall structure was detected after 24 h of conditioning. The swelling of the fibers implicated an increase in surface area that could improve cell infiltration and migration through the 3D electrospun fibrous membrane. Similar results were found for mean roughness measurements and adhesion force. Roughness data were acquired by manually applying a rectangular region of interest (ROI) box of 1 × 1 μm^2^ on the row AFM image. The histograms (Figure 3) show an increase of surface roughness, from 56.8 ± 14.7 nm up to 157.9 ± 25.48 nm, as the SCM treatment went on and an increase from 56.8 ± 14.7 nm up to 102.3 ± 8.7 nm in the PBS. 

**Figure 4:**
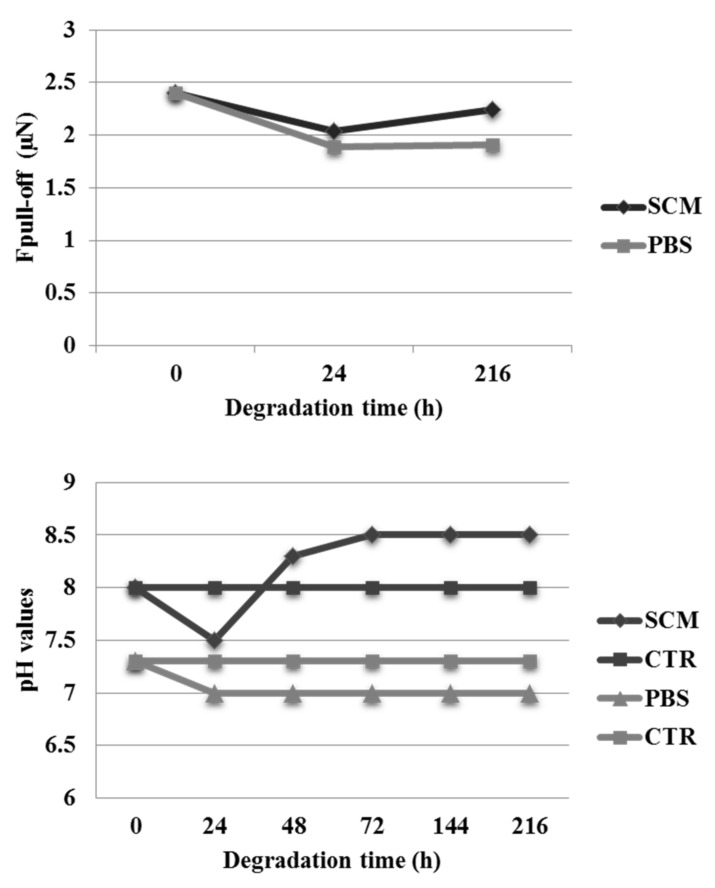
Adhesion force measurements and pH analyses.

The maximum adhesion force, obtained from force–displacement curves as the pull-off force with which detachment between the tip and the sample occurred, was estimated through AFM force spectroscopy for days 1 and 9 of each treatment. The graph showed (Figure 4—first row) a decrease from 2.40 ± 0.49 μN to 2.04 ± 0.52 μN and then to 1.89 ± 0.70 μN in the adhesion force for the SCM and PBS conditioning after 24 h. At longer exposures (9 days), a slight increase up to 2.25 ± 0.61 μN for SCM and to 1.91 ± 0.71 μN for PBS was observed compared to the sample at 24 h but not compared to the control. This result suggests that an aqueous-based biological medium composition, such as SCM, may influence surface properties affecting the different hydrolytic degradations of PCL–gelatin blends (Figure 4—first row). During treatment, changes in pH values in culture medium and in PBS were also evaluated. No changes in pH values were detected for the glass slides without the electrospun fiber (CTR) during conditioning. On the contrary, in the presence of electrospun fibers, pH increased from 8 up to 8.5 after nine days of incubation in SCM treatment according to gelatin release trend, while less evident changes in pH values were recognized during the treatment in PBS. In both experiments, after 24 h, slight decreases in pH value from 8 to 7.5 for SCM and from 7.3 to 7 for PBS treatment were observed. These results suggest that degradation of the fibers in PBS occurs within 24 h, while in the treatment in SCM the degradation rate is slower (Figure 4—second row). Our findings confirm that the medium composition influences the surface properties of the scaffolds causing a different hydrolytic degradation of gelatin.

## 3. Discussion

The understanding and the control of cell/biomaterial interactions is crucial to address cell behavior in terms of proliferation and late activities [27]. It is well-known that bio-inspired scaffolds may variously support the response of cells during in vitro culture. When cells do not attach, they will likely undergo apoptosis and die. Cells can adhere, but they do not spread, thus incurring the same fate. Sometimes, adherent cells may also form fibrotic scar tissue, and investigation on cell attachment can help to prevent this behavior [28,29]. In the latter case, in fact, the mechanisms of cell/scaffold interaction may only be partially controlled in vitro, and the formation of the ECM may be guided in response to specific chemical and topographical cues properly included into the matrix.

BEFs were deeply investigated as candidates mimicking the 3D architecture of natural ECMs. Besides the major shortcoming of gelatin related to its fast dissolution in aqueous media, it has been demonstrated that gelatin, in combination with long-term degradable polymers such as PCL, can form instructive scaffolds—i.e., BEFs—able to exert specific stimuli to cells, thus reproducing the local 3D microenvironment [16,30,31,32]. In particular, in vitro degradation of gelatin macromolecules from BEF fibers may be dynamically recognized by cells during the gradual release from the scaffold. Indeed, the hydrophilic functionalities of gelatin intrinsically enhanced cell adhesion and differentiation, thus providing an active signal of surfaces recognition. The hydrolytic degradation of protein did not significantly compromise the scaffold morphology and fiber integrity. However, an increase in surface roughness as well as a decrease in fiber diameter could potentially influence the interaction mechanisms of cell receptors with the substrate. Hence, AFM analysis may be a powerful means of learning about a cell material’s interaction mechanisms, providing an accurate evaluation of changes in surface roughness as a function of degradation conditions in a simulated biological medium.

Adhesion and roughness analyses, assessed via AFM, proved to be ideal parameters for characterizing mechanical contacts between cell and scaffolds. Recently, a wide number of studies have proposed adhesion force measurements as a quantitative method of evaluating cell attachment to biomaterial as a function of contact time in order to determine a scaffold cytocompatibility [33]. Our findings demonstrate that gelatin release mainly induced a peculiar roughness on the fiber surface, positively influencing local mechanisms of cell adhesion and spreading, with potential effects on the in vitro regeneration process. Moreover, the presence of chemically labile polymer phases, bio-naturally available to the local microenvironment is also really attracting as a vehicle of bioactive macromolecules to support specific cell activities [34,35]. Indeed, gelatin may locally guide the release of molecular cues, also thanks to the use of physical or chemical post-treatments able to modulate the chemical stability of protein in the culture media [36].

Noteworthy recent studies underline how a proper measurement of adhesion and roughness variation can be associated to a pathological state of cells, if compared with healthy ones from the same patient [19,37]. For example, it is well recognized that stiffness, roughness, and morphology play a crucial role on the adhesion and spreading of specific cell lines, thus providing a deep understanding of the interaction between the cell activities and the physicochemical properties of the forming tissue at the implant site [38,39,40]. Accordingly, a peculiar surface characterization at the nano scale performed with advanced AFM tools could be used for a preliminary screening in preclinical trials, i.e., by an investigation of ex vivo explanted cell morphology, or changes in main properties of a cellularized scaffold after the implantation. 

In perspective, the proposed experimental approach could be routinely used prior to the clinical practice by providing predictive information for the investigation of undiscovered biological mechanisms, relevantly contributing to the definition of new in vitro models. 

## 4. Materials and Methods

### 4.1. Materials

PCL (MW 65 kDa, Sigma Aldrich, Milan, Italy), gelatin (Type B from bovine skin, ~225 Bloom), and 1,1,1,3,3,3-hexafluoro-2-propanol (HFP) were purchased from Sigma Aldrich (Milan, Italy). All chemicals and reagents employed in this study were of analytical grade. 

### 4.2. Scaffold Preparation

PCL/HFIP (0.05 g/mL) and gelatin/HFIP (0.05 g/mL) solutions were separately prepared under magnetic stirring. Both solutions were then mixed to obtain a unique solution of PCL/gelatin (50/50 *wt*/*wt*) in HFIP with a polymer concentration of 0.1 g/mL. The solution was dispensed from a 1 mL syringe connected to a hypodermic needle (18 Ga). Different working parameters were selected to optimize the final morphology of fibers: a voltage of 13 kV, a flow rate of 0.5 mL/h, and a distance of 13 cm between the needle and the collector. All electrospun fibers were directly collected on a glass substrate for 1 min using a commercially available electrospinning setup (Nanon01, MECC, Fukuoka, Japan) under a controlled humidity (ca. 40–50%) and temperature (ca. 20–22 °C).

### 4.3. In Vitro Degradation

For degradation study, electrospun membranes were conditioned at 37 °C and 5% carbon dioxide (CO_2_) in simulated culture medium (SCM, i.e., Eagle’s alpha minimum essential medium without protein serum) and in phosphate buffer saline (PBS)—used as control—for 1, 2, 3, 6, and 9 days. The solutions were replaced every 3 days. After 1, 2, 3, 6, and 9 days of exposure, samples of each composition were removed from each solution and washed twice with PBS and then allowed to dry, under a chemical fume hood, before AFM analysis. 

### 4.4. AFM Analyses

AFM analysis allowed for the investigation of gelatin depletion directly from the fiber surface. For the degradation study, electrospun membranes were conditioned at 37 °C and 5% CO_2_ in simulated culture medium (SCM, i.e., Eagle’s alpha minimum essential medium without protein serum) and in PBS for 1, 2, 3, 6, and 9 days. After 1, 2, 3, 6, and 9 days of exposure, samples of each composition were removed from each solution and washed twice with PBS and then allowed to dry before analyses. All imaging was performed in tapping mode (TM-AFM) in air and at room temperature (25 °C). An RTESPA silicon probe (Bruker Corporation, Santa Barbara, CA, USA) with a rotated tip with a radius of 8 nm was used for the characterization. AFM analyses were performed at moderate tapping, according to the Brandsch et al. theory on tapping conditions [38,39,40]. Indeed, the γ_sp_ (the attenuation ratio of the set point amplitude to the free oscillation) was set in a range between 0.4 and 0.7 in order to optimize tip/sample interaction [40]. To calibrate the spring constant of the cantilever before each experiment, the thermal tune calibration method was employed [41]. The resonant frequency and the spring constant of the cantilever were found to fall in their nominal ranges (300–400 kHz and 40–80 N m^−1^). For all the measurements, the scan rate was 0.3 Hz, while the height image scan size was 50 × 50 μm^2^, unless otherwise mentioned. The average diameter was determined by measuring 10 representative fibers on the height AFM image, using NanoScope Analysis data processing software (1.40, Bruker Corporation, Santa Barbara, CA, USA). On the same type of images the cross-section analyses were also performed. The roughness Rq (the root-mean-square height of the surface) was also calculated using AFM software (NanoScope Analysis data processing software 1.40, Bruker Corporation, Santa Barbara, CA, USA). The roughness data was acquired by manually applying a rectangular region of interest (ROI) box of (1 × 1) µm^2^ along the fiber axis at each time point during the degradation experiment [26,42]. In order to obtain an average value of the roughness Rq, the measurements were repeated several times. All results were reported as mean ± standard deviation (SD). The adhesion interaction between the AFM tip and the sample was determined via force spectroscopy AFM curves, and a baseline adjustment was performed before calculating the tip–surface distance. The cantilever deflection was monitored as the tip approaches, contacts and then withdraws from the samples. The calibrated spring constant was used to convert cantilever deflection into force for adhesion analyses. Force-displacement curves were collected at multiple points on the fiber surface and the mean values of all points was considered. The maximum adhesion force, obtained from force–displacement curves as the pull-off force with which detachment between the tip and the sample occurred, was estimated through AFM force spectroscopy after 1 and 9 days of each treatment. 

### 4.5. pH Analysis

During treatment, changes in pH values in SCM and in PBS were also evaluated. The pH value of residual solution in each multiwall plate was collected and measured by pH test strips at each time point to observe whether the pH variation would contribute to gelatin hydrolysis and degradation. To evaluate the pH stability of the SCM and PBS in the incubator, all measurements were compared with controls obtained with glass slides without the electrospun fibers in SCM and PBS, after 1, 2, 3, 6, and 9 days of incubation.

## 5. Conclusions

AFM analysis has been used to estimate gelatin loss from fiber surfaces in SCM and PBS treatments. Results showed a progressive slowing of the hydrolytic degradation of protein that did not significantly alter the fibrous structure. Increases in surface roughness as well as a decrease in fiber diameter were detected. In this context, gelatin release coupled with improved roughness of fibers surface should similarly occur in cell culture, positively influencing the basic mechanisms of cell adhesion and spreading, which better mimics the real microenvironment of natural tissues. Our innovative experimental approach, based on AFM, to evaluate gelatin depletion mechanisms in selected culture media could fill existing gaps in knowledge about the understanding of cell/material interaction mechanisms, opening new opportunities for the definition of personalized 3D models to predict in vivo tissue regeneration.
